# Hydroxytyrosol prevents reduction in liver activity of Δ-5 and Δ-6 desaturases, oxidative stress, and depletion in long chain polyunsaturated fatty acid content in different tissues of high-fat diet fed mice

**DOI:** 10.1186/s12944-017-0450-5

**Published:** 2017-04-11

**Authors:** Rodrigo Valenzuela, Francisca Echeverria, Macarena Ortiz, Miguel Ángel Rincón-Cervera, Alejandra Espinosa, María Catalina Hernandez-Rodas, Paola Illesca, Alfonso Valenzuela, Luis A. Videla

**Affiliations:** 1 Nutrition Department, Faculty of Medicine, University of Chile, Independencia 1027, Casilla, 70000 Santiago 7, Chile; 2 Lipid Center, Institute of Nutrition and Food Technology (INTA), University of Chile, Santiago, Chile; 3 Medical Technology Department, Faculty of Medicine, University of Chile, Santiago, Chile; 4 Biochemistry Department. Faculty of Biochemistry, University of Litoral, Santa Fe, Argentina; 5 Molecular and Clinical Pharmacology Program, Institute of Biomedical Science, Faculty of Medicine, University of Chile, Santiago, Chile

**Keywords:** Hydroxytyrosol, Oxidative stress, Antioxidant capacity, Δ5/Δ6 Desaturase activity, LCPUFA, Liver and extrahepatic tissues

## Abstract

**Background:**

Eicosapentaenoic acid (EPA, C20:5n-3), docosahexaenoic acid (DHA, C22:6n-3) and arachidonic acid (AA, C20:4n-6) are long-chain polyunsaturated fatty acids (LCPUFAs) with relevant roles in the organism. EPA and DHA are synthesized from the precursor alpha-linolenic acid (ALA, C18:3n-3), whereas AA is produced from linoleic acid (LA, C18:2n-6) through the action of Δ5 and Δ6-desaturases. High-fat diet (HFD) decreases the activity of both desaturases and LCPUFA accretion in liver and other tissues. Hydroxytyrosol (HT), a natural antioxidant, has an important cytoprotective effects in different cells and tissues.

**Methods:**

Male mice C57BL/6 J were fed a control diet (CD) (10% fat, 20% protein, 70% carbohydrates) or a HFD (60% fat, 20% protein, 20% carbohydrates) for 12 weeks. Animals were daily supplemented with saline (CD) or 5 mg HT (HFD), and blood and the studied tissues were analyzed after the HT intervention. Parameters studied included liver histology (optical microscopy), activity of hepatic desaturases 5 and 6 (gas-liquid chromatography of methyl esters derivatives) and antioxidant enzymes (catalase, superoxide dismutase, glutathione peroxidase, and glutathione reductase by spectrophotometry), oxidative stress indicators (glutathione, thiobarbituric acid reactants, and the antioxidant capacity of plasma), gene expression assays for sterol regulatory element-binding protein 1c (SREBP-1c) (qPCR and ELISA), and LCPUFA profiles in liver, erythrocyte, brain, heart, and testicle (gas-liquid chromatography).

**Results:**

HFD led to insulin resistance and liver steatosis associated with SREBP-1c upregulation, with enhancement in plasma and liver oxidative stress status and diminution in the synthesis and storage of n-6 and n-3 LCPUFAs in the studied tissues, compared to animals given control diet. HT supplementation significantly reduced fat accumulation in liver and plasma as well as tissue metabolic alterations induced by HFD. Furthermore, a normalization of desaturase activities, oxidative stress-related parameters, and tissue n-3 LCPUFA content was observed in HT-treated rats over control animals.

**Conclusions:**

HT supplementation prevents metabolic alterations in desaturase activities, oxidative stress status, and n-3 LCPUFA content in the liver and extrahepatic tissues of mice fed HFD.

**Electronic supplementary material:**

The online version of this article (doi:10.1186/s12944-017-0450-5) contains supplementary material, which is available to authorized users.

## Background

Linoleic acid (LA, C18:2n-6) and alpha-linolenic acid (ALA, C18:3n-3) are essential polyunsaturated fatty acids (PUFA) for humans and other mammals because they lack the enzymatic machinery to synthesize these fatty acids (FAs) [1]. These PUFAs have important biochemical and physiological functions in the body [2], in addition of being the metabolic precursors of n-6 and n-3 long-chain polyunsaturated fatty acids (LCPUFAs) [3]. LA is the precursor of n-6 LCPUFAs including arachidonic acid (AA, C20:4 n-6), the main metabolic product due to its relevant role in the development of visual and central nervous system and in the control of vascular homeostasis and inflammatory responses [4]. ALA is the precursor of the n-3 LCPUFAs eicosapentaenoic acid (EPA, C20:5n-3) and docosahexaenoic acid (DHA, C22:6n-3), metabolic products that have a number of functions in the body [5]. EPA is involved in the control of vascular homeostasis and resolution of the inflammatory responses [6], whereas DHA has a key role in brain and visual development [7] and in the protection of central and peripheral nervous system [8]. Synthesis of n-6 and n-3 LCPUFA from LA and ALA occurs mainly in the liver through a complex process of elongation and desaturation reactions to yield AA, EPA, and DHA [9], Δ-5 and Δ-6 desaturase representing the most relevant enzymes in LCPUFA biosynthesis, with Δ-6 desaturase being the limiting enzyme of the process [10]. The activity and the expression of both desaturases are regulated by hormones such as insulin and estrogens and by the intracellular redox state [11, 12], and are subjected to polymorphisms resulting in alteration of tissue n-6 and n-3 LCPUFA levels [13]. A significant decrease in the activity of Δ-5 and Δ-6 desaturases has been reported in pathological conditions such as nonalcoholic liver steatosis in humans [14] and in mice subjected to a high-fat diet (HFD) [15], together with a depletion of tissue levels of n-6 and n-3 LCPUFA. Such effects may be mediated by oxidative stress of nutritional origin and by development of insulin resistance [14, 15].

Hydroxytyrosol (HT) is a polyphenol with antioxidant properties found in extra virgin olive oil (EVOO), which together with other components such as polyphenols (for instance tyrosol and oleuropein) and flavonoids support the relevant healthy properties of EVOO [16]. These include the cardio-protective, anti-inflammatory, anti-cancer, and antimicrobial actions of EVOO [16], characteristics that qualify EVOO as a healthy food. HT has shown protective effects at cellular and tissue levels, preventing endothelial cells from damage induced by reactive oxygen species (ROS) [17] and reducing endothelial damage and atherogenic injuries [18]. Different studies have demonstrated the protective effect of HT by (i) preventing LDL oxidation; (ii) inhibiting platelet aggregation; (iii) attenuating mitochondrial abnormalities, with HFD-induced metabolic syndrome prevention [19], and (iv) producing anti-inflammatory effects in association with a decreased activity of the enzymes cyclooxygenase 1 (COX1) and COX2 [20]. One of the main mechanisms underlying the cytoprotective properties of HT is the regulation of different signaling pathways associated to the intracellular redox state [21]. Given this background, the objective of this study was to evaluate the protective effects of HT on the changes induced by a HFD on (i) liver Δ-5 and Δ-6 desaturase enzyme activity and mRNA expression; (ii) plasma and liver oxidative stress-related parameters; and (iii) n-6 and n-3 LCPUFA deposition in liver and extrahepatic tissues of mice fed a HFD.

## Methods

### Animal preparation and supplementation with HT

Weaning male C57BL/6 J mice weighing 12–14 g (Bioterio Central, ICBM, Faculty of Medicine, University of Chile) were randomly assigned to each experimental group (four) and allowed free access to a control diet (CD) or a HFD. CD (expressed as % total calories) was 10% Kcal as fat, 20% Kcal as protein, and 70% Kcal as carbohydrate, with a caloric value of 3.85 kcal/g and free of EPA and DHA. HFD was 60% Kcal as fat, 20% Kcal as protein, and 20% Kcal as carbohydrate, with a caloric value of 5.24 kcal/g and free of EPA and DHA (Research Diet INC, Rodent Diet, Product data D12450K and 12492, USA). Fatty acid composition of CD and HFD was previously published [15]. Compositions of the diets are shown in Additional file 1: Table S1. Animals were housed on a 12 h light/dark cycle from day 1 to 84 (12 weeks) and were provided water *ad libitum*. Supplemented groups received 5 mg HT/day (elaVida^TM^, DSM Nutritional Products Company, Nederland) by oral administration, and control groups received an isovolumetric amount of saline, thus comprising four experimental groups: (a) CD, (b) CD + HT, (c) HFD, and (d) HFD + HT. Weekly controls of body weight and diet intake were performed through the whole period. At the end of the 12th week, animals were fasted (6–8 h), anesthetized with isoflurane, and blood samples were obtained by cardiac puncture for the determination of aspartate transaminase (AST), alanine transaminase (ALT), glucose, insulin, triacylglycerols, total cholesterol, LDL-cholesterol, HDL-cholesterol, thiobarbituric acid reactants (TBARs), and antioxidant capacity. Blood, liver, brain, heart, and testicle samples were frozen in liquid nitrogen for the determination of FA profiles. Liver samples were also fixed in phosphate-buffered formalin, embedded in paraffin, stained with haematoxylin-eosin and analysed by optical microscopy in a blind fashion for describing the presence of steatosis. The degree of steatosis was graded based on an established according to Brunt et al. [22]. Macrovesicular steatosis was graded on a scale 0–3 based on the percentage of hepatocytes affected in each specimen. The score was 0 for none; 1 for up to 33%; 2 for 33–66%; and 3 for more than 66%.

### Measurements of serum parameters and fat content in liver

Serum glucose (mM), total cholesterol (mg/100 mL), LDL-cholesterol (mg/100 mL), HDL-cholesterol (mg/100 mL) and triacylglycerol levels (mg/dL) were measured using specific diagnostic kits (Wiener Lab, Argentina). A commercial immunoassay kit for mice serum insulin assessment (μU/mL) was used, according to the manufacturer's instructions (Mercodia, Uppsala, Sweden). Insulin resistance was estimated by the homeostasis model assessment method (HOMA) [fasting insulin (μU/mL) × fasting glucose (mM)/22.5] [23]. Serum AST and ALT activities (units/L) were measured using specific diagnostic kits (Biomerieux SA, Marcy L’Etoile, France).

### Assay for oxidative stress-related parameters in liver and plasma

Livers from anesthetized animals were perfused *in situ* with a cold solution containing 150 mM KCl and 5 mM Tris (pH 7.4) to remove blood for glutathione assessments. Reduced glutathione (GSH) and glutathione disulfide (GSSG) contents were assessed with an enzymatic recycling method [24]. Specific kits (Cayman Chemical Company, Ann Harbor, MI, USA) were used to measure contents of TBARs in liver and plasma and the antioxidant capacity of plasma according to the manufacturer´s instructions.

### Determination of liver ∆-5 and ∆-6 desaturase activities

Liver samples frozen in liquid nitrogen (500 mg) were homogenized in a buffer solution pH 7.9 containing 10 mmol/L HEPES, 1.0 mmol/L EDTA, 0.6% Nonidet P-40, 150 mmol/L NaCl, and protease inhibitors (1 mmol/L phenylmethylsulfonyl fluoride, 1 μg/mL aprotinin, 1 μg/mL leupeptin, and 1 mmol/L orthovanadate). Liver homogenates were centrifuged at 4 °C, first at 2,000 g for 30 s, followed by centrifugation of the supernatants at 5,000 g for 5 min, and finally at 100,000 g for 60 min, to obtain the extracts for the assessment of desaturase activities. Δ-5 desaturase activity was determined by the amount of dihomo-gamma-linolenic acid (DHGLA, C20:3n-6) converted to AA. Δ-6 desaturase activity was obtained by measuring the amount of gamma-linolenic acid (GLA, C18:3n-6) produced from LA, using albumin-bound FA precursors (DHGLA and LA) [25]. Desaturase activity was assayed using 1 mL of incubation medium containing 4 μmol ATP, 0.1 μmol coenzyme-A, 1.28 μmol NADPH, 2.42 μmol *N*-acetylcysteine, 0.5 μmol nicotinamide, 5 μmol MgCl_2_, 62.5 μmol NaF, and 62.5 μmol phosphate buffer pH 7, supplemented with 100 nmol albumin-bound FA precursor and 1 mg protein of cytosolic extract in a total volume of 100 μL, incubated at 37 °C for 30 min with shaking. Δ-5 and Δ-6 desaturase assays were conducted simultaneously. The reaction was stopped by adding 6 mL of a methanol:chloroform mixture (2:1 v/v). Heptadecanoic acid (17:0; purity ≥ 99%) was added (20 μg) as internal standard. To determine the levels of products or precursors achieved after incubation, lipids were extracted and derivatized to FA methyl esters (FAME), which were analyzed by gas–liquid chromatographic as described previously [26]. FAME peaks were identified and quantified by comparison with a FAME standard mix (Nu-Chek Prep Inc, Elysian MN, USA). Δ-5 and Δ-6 desaturase activities were measured as net increase in DHGLA and GLA production, respectively, from the gas-liquid chromatography results and calculated from the differences between baseline values and those obtained after 30 min incubation. Results were expressed as nmol∙mg protein^−1^∙min^−1^.

### Gene expression assays

Total RNA was isolated from liver samples using Trizol (Invitrogen, Paisley, United Kingdom), according to the supplier’s protocols. Purified RNA (2 μg) was then treated with DNasa (DNA free kit; Ambion, Austin, TX, USA) and used to generate first-strand cDNA with M-MLV reverse transcriptase (Invitrogen), using random hexamers (Invitrogen, Paisley, United Kingdom) and dNTP mix (Bioline, London, United Kingdom), according to the manufacturer`s protocol. The resultant cDNA was amplified with specific primer for mice in a total volume of 10 μL. Gene specific primer sequences used are shown in Additional file 1: Table S2. Primers were optimized to yield 95%-100% of reaction efficiency with PCR products by development in agarose gel to verify the correct amplification length. Real Time PCR was performed in a Strategen Mx3000P System (Agilent Technologies, California, USA) following the manufacturer`s recommendation (Applied Biosystems, Foster City, CA, USA). All the expression levels of the target genes under study were normalized by the expression of β-actin as internal control (Applied Biosystems, Foster City, CA, USA). Fold changes between groups was calculated by the 2(^-ΔΔCt^) method.

### Assessment of liver sterol regulatory element-binding protein 1c (SREBP-1c) DNA-binding activity

Nuclear extracts from liver tissue (left lobe) were obtained using a commercial extraction kit (Cayman Chemical Company, Item 10011223, Ann Arbor, MI, USA). SREBP-1c DNA-binding activity was assessed with a commercial ELISA kit (Cayman Chemical Company, Item 10010854) and according to the manufacturer´s instructions. Values were expressed as percentage of SREBP-1c DNA-binding with respect to a positive control provided by the ELISA kit.

### Determination of liver catalase, superoxide dismutase, glutathione peroxidase, and glutathione reductase activities

Liver samples were homogenized in three volumes of 30 mM phosphate buffer, pH 7.4, containing EDTA (1 mM) and sucrose (250 mmol). After centrifugation for 10 min at 1200 g and 4 °C, one aliquot of the supernatant was used for the determination of both catalase (CAT) and superoxide dismutase (SOD) activities. Another aliquot was centrifuged at 100,000 g for 60 min at 4 °C to carry out glutathione peroxidase (GPX) and glutathione reductase (GR) assays according to Chow [27]. CAT activity was measured according to Lück [28]. Enzyme unit is defined as the amount of oxygen liberated from a hydrogen peroxide solution in 100 s at 25 °C. Assessment of SOD activity was carried out with a commercial assay kit (Cayman Chemical Company, Ann Arbor, MI, USA) according to the manufacturer´s instructions. GPX activity was determined using hydrogen peroxide as substrate according to the method of Plagia and Valentine [29]. Enzyme activity was evaluated at 340 nm by measuring the decrease in the absorbance of NADPH. Enzyme unit is defined as the number of μmoles of NADPH oxidized per min at 20 °C. GR activity was determined by the method of Horn [30]. Enzyme unit is defined as the amount of enzyme that reduces 1 μmol of GSSG per min at pH 6.6 and 25 °C.

### Lipid extraction and fractionation

Quantitative extraction and separation of total lipids from liver, erythrocytes, brain, heart, and testicle was carried out according Bligh and Dyer [31]. Erythrocytes and tissue samples were homogenized in ice-cold chloroform/methanol (2:1 v/v) containing 0.01% butylated hydroxytoluene (BHT). Total lipids from erythrocytes were extracted with chloroform/isopropanol (2:1 v/v). Phospholipids from liver, erythrocytes, brain, heart, and testicle were separated from total lipid extracts by thin layer chromatography (TLC) on silica gel plates (aluminium sheets 20x20 cm, silica gel 60 F-254 (Merck, Santiago, Chile), using hexane/diethyl ether/acetic acid (80:20:1 v/v/v) as mobile phase. After development and solvent evaporation, lipid spots were visualized by exposing the plates to a Camag UV (250 nm). The solvent system allows the separation of phospholipids, cholesterol, triacylglycerols and cholesterol ester according to their relative mobility. Phospholipid spots were extracted from the silica with chloroform/methanol (2:1 v/v), according to Ruiz-Gutierrez et al. [32].

### Fatty acid analysis

FAME from liver, erythrocytes, brain, heart, and testicle phospholipids were prepared with boron trifluoride according to Morrison and Smith [33], and a sodium hydroxide solution (0.5 N) in methanol. Phospholipids were extracted from the silica gel spots with 15 mL of chloroform/methanol/water (10:10:1) and solvents were then evaporated under nitrogen stream prior to derivatization of phospholipids to produce FAMEs. Samples were cooled and extracted with 0.5 mL of hexane. FAMEs were separated and quantified by gas-liquid chromatography in an Agilent Hewlett-Packard equipment (model 7890A, CA, USA) using a capillary column (Agilent HP-88, 100 m x 0.250 mm; I.D. 0.25 μm) and a flame ionization detector (FID). Injector temperature was set at 250 °C and FID temperature at 300 °C. Oven temperature was initially set at 140 °C and programmed to increase to 220 °C at a rate of 5 °C/min. Hydrogen was used as the carrier gas (35 cm/s flow rate) and the inlet split ratio was set at 20:1. Identification and quantification of FAMEs were achieved by comparing the retention times and the peak area values (%) of the unknown samples with those of a commercial lipid standard (Nu-Chek Prep Inc, Elysian MN, USA). C23:0 was used as internal standard (Nu-Chek Prep Inc, Elysian MN, USA) and a Hewlett-Packard Chemstation (Palo Alto, CA, USA) data system was used for processing.

### Statistical analysis

Statistical analysis was performed with GraphPad Prism 6.0 software (GraphPad Prism, Inc., San Diego, USA). The values shown represent the mean ± SEM for each experimental group. Evaluation of normality data distribution was performed using the Shapiro-Wilk test. Assessment of the statistical significance of differences between mean values was performed by one-way ANOVA and the Newman-Keuls´ test. A *p* < 0.05 was considered significant. Analysis of association between different variables was carried-out using the Pearson correlation coefficient.

## Results

### General physiological parameters (A), food intake (B), serum parameters (C), insulin resistance parameters (D), and liver parameters (E) of CD and HFD fed mice receiving HT supplementation (Table 1)

**Table 1:** General and biochemical parameters in control mice and high-fat diet fed mice subjected to hydroxytyrosol (HT) supplementation

	Groups			
	Control diet (CD)		High-fat diet (HFD)	
	Saline (a)	HT (b)	Saline (c)	HT (d)
A. General parameters				
Initial body weight (g)	13.5 ± 0.6	14.0 ± 0.7	14.0 ± 0.5	13.2 ± 0.8
Final body weight (g)	34.4 ± 2.7^c,d^	36.2 ± 3.1^c,d^	46.8 ± 4.5^a,b^	42.5 ± 3.2^a,b^
Liver weight (g)	1.19 ± 0.2	1.21 ± 0.4	1.25 ± 0.3	1.24 ± 0.4
Liver weight (g)/ final body weight (g) ratio	0.034 ± 0.01^c,d^	0.033 ± 0.01^c,d^	0.026 ± 0.005^a,b,d^	0.029 ± 0.01^a,b,c^
B. Food and Energy intake				
Food intake (g/day)	2.4 ± 0.5	2.3 ± 0.4	2.7 ± 0.6	2.6 ± 0.3
Energy intake (kcal/day)	9.22 ± 0.8^c,d^	8.84 ± 0.7^c,d^	14.2 ± 1.4^a,b^	13.6 ± 1.3^a,b^
C. Lipid parameters				
Triacylglycerols (mg/dL)	130.5 ± 7.2^c,d^	120.6 ± 6.5^c,d^	180.6 ± 11.5^a,b,d^	156.3 ± 8.4^a,b,c^
Total cholesterol (mg/dL)	73.5 ± 8.5^c,d^	68.7 ± 6.9^c,d^	126.5 ± 15.3^a,b,d^	103.6 ± 9.6^a,b,c^
LDL-cholesterol (mg/dL)	44.3 ± 3.8^c,d^	40.2 ± 3.3^c,d^	78.6 ± 6.5^a,b,d^	63.5 ± 5.4^a,b,c^
HDL-cholesterol (mg/dL)	26.9 ± 3.4^c,d^	24.7 ± 2.9^c,d^	44.9 ± 4.8^a,b^	36.3 ± 5.7^a,b^
D. Insulin resistance				
Fasting glucose (mg/dL)	123.5 ± 5.6^c,d^	119.4 ± 4.9^c,d^	235.5 ± 29.4^a,b,d^	184.6 ± 16.5^a,b,c^
Fasting insulin (units/mL)	5.47 ± 0.7^c,d^	5.19 ± 0.8^c,d^	18.4 ± 1.7^a,b,d^	12.2 ± 1.1^a,b,c^
HOMA	1.25 ± 0.3^c.d^	1.17 ± 0.2^c,d^	8.92 ± 1.0^a,b,d^	6.45 ± 0.6^a,b,d^
E. Serum transaminases				
AST (U/L)	139.3 ± 17.8	142.7 ± 20.3	171.5 ± 28.5	148.6 ± 23
ALT (U/L)	78.6 ± 9.8	65.2 ± 6.4	80.2 ± 10.7	75.3 ± 5.6

Values represent means ± SEM for 12–14 mice per experimental group. Significant differences between the groups are indicated by the letter identifying each group (one-way ANOVA and Newman-Keuls´ post-test; *p* < 0.05)

**Fig. 1:**
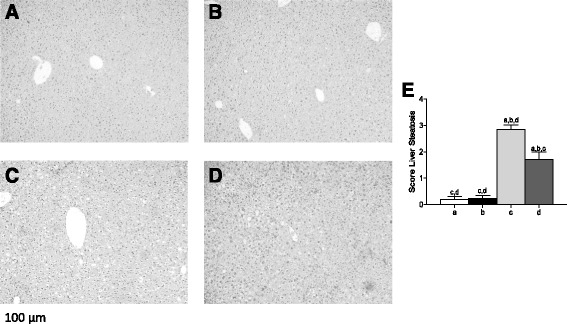
Liver histology in control and high-fat diet (HFD) fed mice receiving hydroxytyrosol (HT) supplementation. Representative liver sections are from **a** CD diet, **b** CD + HT, **c** HFD, **d** HFD + HT (haematoxylin-eosin liver sections from of 8–10 animals per experimental group; original magnification x10) and (**e**) Score Liver Steatosis. Significant differences between the groups are indicated by the letter identifying each group (one-way ANOVA and Newman-Keuls´ post-test; *p* < 0.05)

HFD produced a significant increment of body weight of animals at the end of the intervention, but HT supplementation did not produce weight modification either in control or in HFD mice. Liver weight was also not modified by HFD nor by HT. However, a significant difference was observed for the ratio liver weight/final body weight. Food intake was similar for both experimental groups, energy intake of HFD group being significantly higher than CD. Supplementation with HT did not generate significant changes in dietary intake. Serum parameters were all modified by HFD compared to control. HT supplementation did not modify these parameters in controls, but significantly reduced triacylglycerol, total cholesterol and LDL-cholesterol in HFD mice, without changes in HDL-cholesterol. Insulin resistance parameters were also drastically modified by HFD, with significant increases in fasting glucose, fasting insulin, and HOMA values. HT supplementation reduced these parameters but not to the levels of the respective controls. Serum AST and ALT transaminases were not modified by the HFD nor HT supplementation. These results demonstrate that HT improved some serum parameters and markers of insulin resistance, without restoration to the levels observed in control animals. Figure 1 shows study of liver morphology indicated the presence of severe micro and macrovesicular steatosis in the HFD group (Fig. 1c), compared to those subjected to CD and CD + HT (Fig. 1a and b), whereas hepatic fat accumulation was recorded as mild in the HFD + HT group (Fig. 1d). Respect to liver score steatosis, this parameter was increased by HFD in mice (Fig. 1e), and attenuated by HT but not to the same values in control groups (Fig. 1e).

### HT supplementation normalizes the changes induced by HFD on liver desaturase activities and desaturase gene expression, SREBP-1c mRNA expression, and SREBP 1-c DNA binding activity

**Fig. 2:**
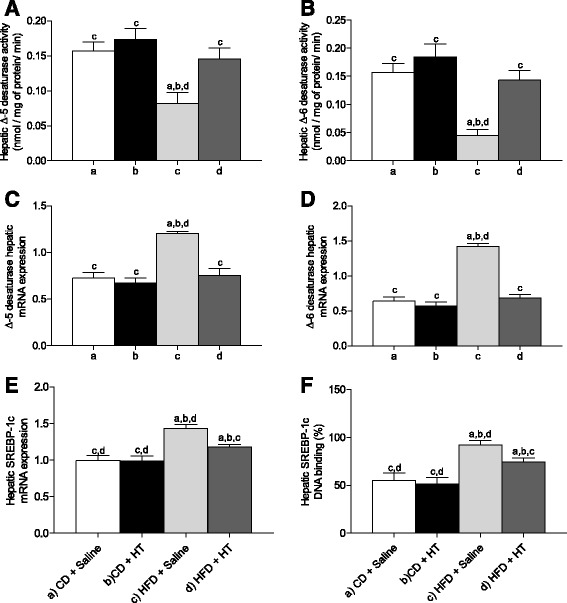
Liver desaturase activities and expression and liver SREBP-1c expression and DNA-binding activity from control mice and high fat diet (HFD) fed mice receiving hydroxytyrosol (HT) supplementation. **a** Δ-5 desaturase activity; **b**, Δ-6 desaturase activity; **c**, Δ-5 desaturase mRNA expression; **d**, Δ-6 desaturase mRNA expression; **e**, SREBP-1c mRNA expression; and **f**, SREBP-1c DNA binding activity. Values represent means ± SEM for 12–14 mice per experimental group. Significant differences between the groups are indicated by the letter identifying each group (one-way ANOVA and Newman-Keuls´ post-test; *p* < 0.05)

Figure 2 shows ∆-5 desaturase (Fig. 2a) and ∆-6 desaturase (Fig. 2b) activities, ∆-6 desaturase (Fig. 2c) and ∆-5 desaturase (Fig. 2d) mRNA expression, SREBP 1c mRNA expression (Fig. 2e) and SREBP 1-c DNA binding activity (Fig. 2f), after HFD and HT supplementation. The activity of both desaturases was strongly reduced by HFD, but recovery to control values was achieved by HT. In contrast, HFD produced a significantly increase of mRNA expression of both desaturases, which returned to control values by HT, a paradoxical effect can be interpreted as a compensatory response to the reduction in the activity of desaturases. SREBP 1-c mRNA expression and SREBP 1-c DNA binding activity were also increased by HFD and reduced by HT, without reaching the control figures. Again, the increased mRNA and DNA binding activity of SREBP 1-c by HFD may be a compensatory mechanism to the reduction in the activity of desaturases. Results show a strong metabolic effect of HFD on parameters related to lipid metabolism and a relevant recovery response after HT supplementation.

### HT supplementation offsets the changes in oxidative stress-related parameters induced by HFD in plasma and liver

**Fig. 3:**
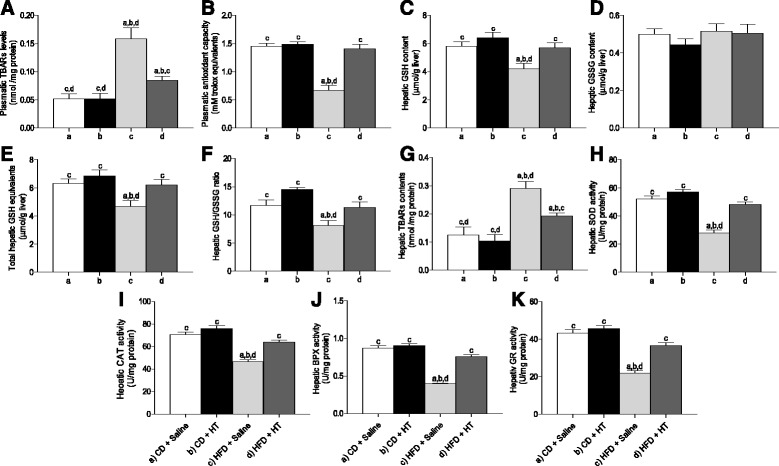
Oxidative stress-related parameters in the liver and plasma from control mice and high-fat diet (HFD) fed mice receiving hydroxytyrosol (HT) supplementation. **a** plasma TBARs; **b** plasma antioxidant capacity; **c**, liver GSH; **d**, liver GSSG; **e**, liver total GSH equivalents (GSH + 2GSSG); **f**, liver GSH/GSSG ratio; **g**, liver TBARs; **h**, liver SOD activity; **i**, liver CAT activity; **j**, liver GPX activity; and **k**, liver GR activity. Values represent means ± SEM for 12–14 mice per experimental group. Significant differences between the groups are indicated by the letter identifying each group (one-way ANOVA and Newman-Keuls´ post-test; *p* < 0.05)

Figure 3 shows oxidative stress-related parameters obtained from mice fed control diet + saline (CD + saline), control diet + HT (CD + HT), HFD + saline, and HFD + HT, namely, plasma TBARs (Fig. 3a), plasma antioxidant capacity (Fig. 3b), liver GSH (Fig. 3c), liver GSSG (Fig. 3d), total GSH equivalents (Fig. 3e), liver GSH/GSSG ratio (Fig. 3f), and liver TBARs (Fig. 3g). Results demonstrate that HFD significantly increased plasma TBARs concomitant with a reduction of plasma antioxidant capacity. HT improved these values reducing TBARs to values close, but not similar, to control values. HT also increased plasma antioxidant capacity, restoring the values found in controls. Liver GSH but not GSSG was reduced by HFD, however, HT restored GSH, total GSH equivalent, and GSH/GSSG ratio to values similar to control. Liver TBARs were also increased by HFD and restored by HT but not to the same values in controls. Results demonstrate an effective protection of HT supplementation against modification of plasma and liver oxidative stress-related parameters induced by HFD.

In addition, Fig. 3 shows the activity of liver SOD (Fig. 3h), CAT (Fig. 3i), PGX (Fig. 3j), and GR (Fig. 3k) after HFD and HT supplementation. The activity of all these enzymes was significantly reduced by HFD. However, HT supplementation restored these activities to values similar to those found in controls. Results demonstrate a major improvement of the antioxidant enzyme potential of the liver by HT supplementation after HFD feeding.

### Correlations between liver ∆-5 and ∆-6 desaturase activities with oxidative stress-related parameters

**Fig. 4:**
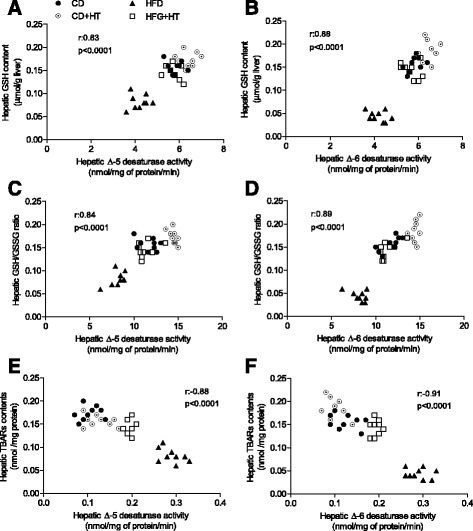
Correlation of ∆-5 and ∆-6 desaturase activities with oxidative stress-related parameters in the liver of control mice and high-fat diet (HFD) fed mice receiving hydroxytyrosol (HT) supplementation. **a** GSH content v/s ∆-5 desaturase activity; **b**, GSH content v/s ∆-6 desaturase activity; **c**, GSH/GSSG ratio v/s ∆-5 desaturase activity; **d**, GSH/GSSG ratio v/s ∆-6 desaturase activity; **e**, TBARs content v/s ∆-5 desaturase activity; and **f**, TBARs content v/s ∆-6 desaturase activity

Figure 4 shows the correlation between liver desaturase activities (Δ-5 and Δ-6) with oxidative stress parameters. A positive correlation is observed for liver GSH content and ∆-5 desaturase activity (Fig. 4a; *r* = 0.85, *p* < 0.0001) and ∆-6 desaturase activity (Fig. 4b; *r* = 0.88, *p* < 0.0001), as well as for hepatic GSH/GSSG ratios and ∆-5 desaturase activity (Fig. 4c; *r* = 0.84, *p* < 0.0001) and ∆-6 desaturase activity (Fig. 4d; *r* = 0.89, *p* < 0.0001). Liver TBARs content exhibited a negative correlation with ∆-5 desaturase activity (Fig. 4e; *r* = −0.88, *p* < 0.0001) and with ∆-6 desaturase activity (Fig. 4f; *r* = −0.91, *p* < 0.0001). These associations indicate that the desaturase activity of the liver is privileged when an elevated anti-oxidative status is present, either through higher GSH levels and GSH/GSSG ratios or lower TBARs contents, suggesting that oxidative stress may affect n-6 and n-3 LCPUFA biosynthesis from the respective precursors. Also, to evaluate the correlation between plasma antioxidant capacity with the activity of liver desaturase enzyme (Δ-5 and Δ-6 was observed a positive correlation into this parameter of redox systemic status with the activity of this enzymes (Δ-5; *r* = 0.88, *p* < 0.0001 and Δ-6; *r* = 0.91, *p* < 0.0001, respectively).

### HT supplementation counteracts the changes in the fatty acid composition of phospholipids from liver, erythrocyte, brain, heart, and testicle induced by HFD

Table 2 shows that, with the exception of LA, most FAs were reduced by the HFD compared to control. HT supplementation did not produce differences in control animals, but FAs such as ALA, AA, EPA, and DHA were significantly increased in HFD group receiving HT to the levels observed in control animals. In HFD animals supplemented with HT, total SFAs were reduced whereas total PUFAs, total LCPUFAs, total n-6 LCPUFAs, and total n-3 LCPUFAs were increased to levels similar to those found in controls (saline or HT). N-6/n-3 LCPUFA ratio was restored to the value found in controls (saline or HT) when HT was incorporated to the HFD. Data show that supplementation with HT avoids the reduction of n-6 and n-3 PUFA and of n-6 and n-3 LCPUFA induced by the HFD in mice, reflecting a protective action of the polyphenol on the FA composition of liver phospholipids.

**Table 2:** Fatty acid composition of liver phospholipids from control mice and high-fat diet fed mice subjected to hydroxytyrosol (HT) supplementation

	Fatty acid composition (g/100 g FAME)			
	Groups			
	Control diet (CD)		High fat diet (HFD)	
	Saline	HT	Saline	HT
Fatty acid	(a)	(b)	(c)	(d)
C16:0	31.2 ± 2.6^c^	28.9 ± 2.1^c^	43.6 ± 4.2^a,b,d^	32.4 ± 3.3^c^
C18:0	4.31 ± 0.6^b^	3.15 ± 0.2^a^	4.62 ± 0.6^b^	4.40 ± 0.4^b^
C18:1n-9	23.4 ± 1.8	25.6 ± 2.3	22.2 ± 1.6	24.8 ± 2.0
C18:2n-6 (LA)	13.3 ± 1.1	14.5 ± 1.3	11.8 ± 1.0	12.1 ± 1.2
C18:3n-6	1.10 ± 0.1	0.97 ± 0.1	0.95 ± 0.05	0.99 ± 0.05
C18:3n-3 (ALA)	1.19 ± 0.1^c^	1.23 ± 0.2^c^	0.89 ± 0.05^a,b,d^	1.09 ± 0.04^c^
C18:4n-3	0.25 ± 0.03^c^	0.27 ± 0.05^c^	0.09 ± 0.02^a,b,d^	0.23 ± 0.04^c^
C20:3n-6	0.27 ± 0.05^c^	0.29 ± 0.04^c^	0.15 ± 0.03^a,b,d^	0.26 ± 0.05^c^
C20:4n-6 (AA)	10.2 ± 0.9^c^	11.5 ± 0.7^c^	5.95 ± 0.4^a,b,d^	10.4 ± 0.8^c^
C20:4n-3	0.16 ± 0.04^c^	0.17 ± 0.05^c^	0.04 ± 0.02^a,b,d^	0.14 ± 0.3^c^
C20:5n-3 (EPA)	1.07 ± 0.1^c^	1.10 ± 0.1^c^	0.32 ± 0.04^a,b,d^	1.05 ± 0.05^c^
C22:5n-6 (DPAn-6)	0.08 ± 0.01^c^	0.09 ± 0.02^c^	0.03 ± 0.01^a,b,d^	0.08 ± 0.01^c^
C22:5n-3 (DPAn-3)	0.09 ± 0.02^c^	0.08 ± 0.01^c^	0.04 ± 0.01^a,b,d^	0.08 ± 0.01^c^
C22:6n-3 (DHA)	4.21 ± 0.2^c^	4.39 ± 0.3^c^	1.95 ± 0.1^a,b,d^	4.12 ± 0.2^c^
Total SFAs	37.5 ± 3.1^c^	36.4 ± 2.9^c^	48.9 ± 4.4^a,b,d^	38.7 ± 3.5^c^
Total MUFAs	26.6 ± 2.2	28.5 ± 1.9	24.4 ± 2.4	26.7 ± 2.0
Total PUFAs	35.9 ± 3.1^c^	35.1 ± 3.6^c^	26.7 ± 2.3^a,b,d^	34.6 ± 3.3^c^
Total LCPUFAs	16.6 ± 1.5^c^	17.9 ± 1.1^c^	8.62 ± 0.5^a,b,d^	16.4 ± 0.9^c^
Total n-6 LCPUFAs	11.0 ± 0.7^c^	12.1 ± 0.8^c^	6.17 ± 0.3^a,b,d^	10.9 ± 0.8^c^
Total n-3 LCPUFAs	5.60 ± 0.3^c^	5.80 ± 0.4^c^	2.45 ± 0.2^a,b,d^	5.50 ± 0.5^c^
n-6/n-3 LCPUFA ratio	1.96 ± 0.1^c^	2.08 ± 0.05^c^	2.51 ± 0.2^a,b,d^	1.98 ± 0.1^c^

Values are expressed as g fatty acid per 100 g FAME and represent the mean ± SEM for *n* = 12–14 mice per experimental group. Significant differences between the groups are indicated by the letter identifying each group (one-way ANOVA and the Newman-Keuls´ post-test; *p* < 0.05). Saturated fatty acids (SFAs) correspond to C12:0, C14:0, C16:0 and C18:0. Monounsaturated fatty acids (MUFAs) correspond to C14:1n-7, C16:1n-7 and C18:1n-9. Polyunsaturated fatty acids (PUFAs) correspond to C18:2n-6, C18:3n-3, C20:4n-6, C20:5n-3, C22:5n-3, and C22:6n-3; n-6 LCPUFAs are C20:4n-6 and C22:5n-3; n-3 LCPUFAs are C20:5n-3, C22:5n-3, and C22:6n-3; n-6/n-3 LCPUFA ratio: C20:4n-6/ (C20:5n-3 + C22:5n-3 + C22:6n-3)

In contrast to that observed in liver tissue, erythrocyte phospholipids showed LA, ALA, and AA levels that were not modified by HFD and no effect of HT was observed, however, the main n-3 LCPUFAs EPA and DHA were significantly reduced by HFD, but restored to the control level by HT (Table 3). A similar effect was observed for other minor n-6 and n-3 LCPUFAs such as DPA (n-6) and DPA (n-3). As a result of the HT-induced recovery of EPA and DHA, total LCPUFAs, total n-3 LCPUFAs, and n-6/n-3 LCPUFA ratios reached values similar to those found in control (Table 3). HT supplementation did not modify the FA level of control mice (saline or HT; Table 3).

**Table 3:** Fatty acid composition of erythrocyte phospholipids from control mice and high-fat diet (HFD) fed mice receiving hydroxytyrosol (HT) supplementation

	Fatty acid composition (g/100 g FAME)			
	Groups			
	Control diet (CD)		High fat diet (HFD)	
	Saline	HT	Saline	HT
Fatty acid	(a)	(b)	(c)	(d)
C16:0	34.5 ± 3.1^c^	31.9 ± 2.9^c^	41.6 ± 3.8^a,b,d^	36.6 ± 3.5^c^
C18:0	3.62 ± 0.3^c,d^	3.45 ± 0.4^c,d^	6.58 ± 0.7^a,b^	5.58 ± 0.6^a,b^
C18:1n-9	24.6 ± 1.9	25.6 ± 2.2	22.9 ± 2.3	24.8 ± 2.5
C18:2n-6 (LA)	9.91 ± 1.2	10.2 ± 0.9	8.66 ± 1.7	9.52 ± 1.5
C18:3n-3 (ALA)	1.21 ± 0.5	1.12 ± 0.3	1.05 ± 0.3	1.05 ± 0.3
C20:4n-6 (AA)	14.6 ± 1.4	15.7 ± 1.6	12.6 ± 1.1	12.4 ± 1.3
C20:5n-3 (EPA)	2.05 ± 0.3^c^	2.19 ± 0.4^c^	0.75 ± 0.03^a,b,d^	1.96 ± 0.2^c^
C22:5n-6 (DPAn-6)	0.16 ± 0.04^c^	0.19 ± 0.05^c^	0.05 ± 0.01^a,b,d^	0.12 ± 0.02^c^
C22:5n-3 (DPAn-3)	0.78 ± 0.05^c^	0.86 ± 0.06^c,d^	0.25 ± 0.01^a,b,d^	0.70 ± 0.04^b,c^
C22:6n-3 (DHA)	4.11 ± 0.3^c^	4.32 ± 0.4^c^	1.98 ± 0.1^a,b,d^	3.98 ± 0.3^c^
Total SFAs	38.8 ± 2.9^c^	36.5 ± 2.6^c,d^	49.7 ± 4.4^a,b^	43.5 ± 3.9^b^
Total MUFAs	26.4 ± 2.5	27.9 ± 2.4	24.5 ± 2.2	24.8 ± 2.7
Total PUFAs	34.8 ± 3.3^c^	35.6 ± 3.6^c^	25.8 ± 2.2^a,b^	31.7 ± 2.6
Total LCPUFAs	22.9 ± 1.7^c^	23.7 ± 2.1^c^	15.8 ± 1.1^a,b,d^	20.2 ± 1.5^c^
Total n-6 LCPUFAs	14.9 ± 1.4	16.0 ± 1.6^c^	12.7 ± 0.9^b^	12.7 ± 1.4
Total n-3 LCPUFAs	8.00 ± 0.7^c^	7.70 ± 0.5^c^	3.10 ± 0.2^a,b,d^	7.50 ± 0.6^c^
n-6/n-3 LCPUFA ratio	1.86 ± 0.2^c^	2.07 ± 0.3^c^	4.10 ± 0.4^a,b,d^	1.69 ± 0.3^c^

Values are expressed as g fatty acid per 100 g FAME and represent the mean ± SEM for *n* = 12–14 mice per experimental group. Significant differences between the groups are indicated by the letter identifying each group (one-way ANOVA and Newman-Keuls´ post-test; *p* < 0.05). The identification of saturated and unsaturated fatty acids and their relationships are shown in Table 2

FA composition of brain phospholipids was strongly modified by HFD, showing a notably response to HT supplementation (Table 4). HFD increased C16:0 and DPA (n-6), but reduced ALA, AA, EPA, DPA (n-3), and DHA; although HT supplementation in HFD fed mice restored these FA to values similar to control (saline and HT). The same result is observed for total PUFAs, total LCPUFAs, total n-6 LCPUFAs, total n-3 LCPUFAs and n-6/n-3 LCPUFA ratios (Table 4). These results demonstrate that brain is a very sensitive tissue to the modification of FA supply and that HT may exert an important protection against modification of FA profiles caused by a HFD.

**Table 4:** Fatty acid composition of brain phospholipids from control mice and high-fat diet (HFD) fed mice receiving hydroxytyrosol (HT) supplementation

	Fatty acid composition (g/100 g FAME)			
	Groups			
	Control diet (CD)		High fat diet (HFD)	
	Saline	HT	Saline	HT
Fatty acid	(a)	(b)	(c)	(d)
C16:0	35.6 ± 3.1^c^	34.3 ± 3.3^c^	47.4 ± 4.6^a,b,d^	37.3 ± 3.6^c^
C18:0	5.95 ± 0.6	5.58 ± 0.4	5.15 ± 0.4	5.75 ± 05
C18:1n-9	19.8 ± 1.6	17.9 ± 1.4	20.4 ± 1.7	19.6 ± 1.8
C18:2n-6 (LA)	3.65 ± 0.2	3.52 ± 0.3	3.12 ± 0.2	3.71 ± 0.4
C18:3n-3 (ALA)	1.30 ± 0.3^c^	1.27 ± 0.2^c^	0.52 ± 0.05^a,b,d^	1.11 ± 0.1^c^
C20:4n-6 (AA)	17.1 ± 1.2^c^	17.8 ± 1.4^c^	11.7 ± 0.7^a,b,d^	15.9 ± 1.6^c^
C20:5n-3 (EPA)	0.82 ± 0.04^c^	0.86 ± 0.03^c,d^	0.51 ± 0.02^a,b,d^	0.76 ± 0.05^c^
C22:5n-6 (DPAn-6)	0.30 ± 0.05^c,d^	0.26 ± 0.05^c,d^	1.33 ± 0.08^a,b,d^	1.10 ± 0.04^a,b,c^
C22:5n-3 (DPAn-3)	0.45 ± 0.04^c^	0.49 ± 0.05^c,d^	0.24 ± 0.02^a,b,d^	0.42 ± 0.03^b,d^
C22:6n-3 (DHA)	11.5 ± 1.2^c^	12.3 ± 1.4^c^	7.02 ± 0.6^a,b,d^	10.9 ± 1.5^c^
Total SFAs	42.5 ± 3.1^c^	40.8 ± 2.9^c^	52.8 ± 3.8^a,b,d^	43.8 ± 3.3^c^
Total MUFAs	21.7 ± 1.9	20.6 ± 1.7	22.5 ± 1.5	21.2 ± 2.0
Total PUFAs	35.8 ± 2.8^c^	38.6 ± 3.0^c^	24.7 ± 2.1^a,b,d^	35.0 ± 3.1^c^
Total LCPUFAs	30.5 ± 2.3^c^	32.6 ± 2.9^c^	21.0 ± 1.8^a,b,d^	29.5 ± 2.5^c^
Total n-6 LCPUFAs	17.6 ± 1.3^c^	18.4 ± 1.4^c^	13.2 ± 1.0^a,b,d^	17.3 ± 1.4^c^
Total n-3 LCPUFAs	12.9 ± 1.0^c^	14.2 ± 1.1^c^	7.80 ± 0.4^a,b,d^	12.2 ± 0.8^c^
n-6/n-3 LCPUFA ratio	1.36 ± 0.2	1.29 ± 0.2	1.69 ± 0.3	1.41 ± 0.2

Values are expressed as g fatty acid per 100 g FAME and represent the mean ± SEM for *n* = 12–14 mice per experimental group. Significant differences between the groups are indicated by the letter identifying each group (one-way ANOVA and Newman-Keuls´ post-test; *p* < 0.05). The identification of saturated and unsaturated fatty acids and their relationships are shown in Table 2

The FA composition of heart phospholipids was also modified by the dietary intervention with HT (Table 5). The increase of SFA such as C16:0, and total SFA produced by HFD, was normalized to values similar to control by HT supplementation. Besides, the reduction of ALA, EPA, DPA (n-6), DPA (n-3), and DHA produced by HFD was also normalized by HT to values close to the control (both saline and HT). Although total n-6 LCPUFA levels were not recovered by HT as observed in other tissues, total n-3 LCPUFAs showed an increase without reaching the values observed in controls. HFD-induced reduction in the n-6/n-3 LCPUFA ratio exhibited a partial recovered by HT (Table 5). Results demonstrate that FA profile of the heart, which is a highly demanding metabolic tissue, is also modified by HFD and positively affected by HT availability.

**Table 5:** Fatty acid composition of heart phospholipids from control mice and high-fat diet (HFD) fed mice receiving hydroxytyrosol (HT) supplementation

	Fatty acid composition (g/100 g FAME)			
	Groups			
	Control diet (CD)		High fat diet (HFD)	
	Saline	HT	Saline	HT
Fatty acid	(a)	(b)	(c)	(d)
C16:0	33.5 ± 2.9^c^	32.8 ± 3.0^c^	42.9 ± 3.4^a,b,d^	34.7 ± 2.7^c^
C18:0	4.84 ± 0.3	4.32 ± 0.5	3.94 ± 0.3	5.11 ± 0.5
C18:1n-9	22.8 ± 2.3	23.9 ± 2.5	22.4 ± 1.9	23.3 ± 2.7
C18:2n-6 (LA)	11.7 ± 1.5	11.6 ± 1.3	11.9 ± 1.6	12.5 ± 1.2
C18:3n-3 (ALA)	1.36 ± 0.1^c,d^	1.44 ± 0.1^c,d^	0.82 ± 0.04^a,b,d^	1.12 ± 0.05^a,b,c^
C20:4n-6 (AA)	11.6 ± 1.4	10.8 ± 1.2	10.90 ± 1.6	11.3 ± 1.7
C20:5n-3 (EPA)	2.82 ± 0.1^c,d^	2.99 ± 0.1^c,d^	0.98 ± 0.04^a,b,d^	1.53 ± 0.05^a,b,c^
C22:5n-6 (DPAn-6)	0.68 ± 0.1^c^	0.75 ± 0.05^c^	0.35 ± 0.02^a,b,d^	0.62 ± 0.04^c^
C22:5n-3 (DPAn-3)	0.72 ± 0.05^c^	0.79 ± 0.1^c^	0.28 ± 0.01^a,b,d^	0.67 ± 0.05^c^
C22:6n-3 (DHA)	5.15 ± 0.3^c^	5.36 ± 0.2^c,d^	2.05 ± 0.1^a,b,d^	4.77 ± 0.2^b,c^
Total SFAs	39.3 ± 3.3^c^	37.8 ± 3.0^c^	47.9 ± 4.1^a,b,d^	40.3 ± 3.8^c^
Total MUFAs	25.6 ± 2.6	26.9 ± 2.4	24.4 ± 2.2	25.6 ± 2.7
Total PUFAs	35.1 ± 3.1^c^	35.3 ± 3.3^c^	28.0 ± 2.5^a,b,d^	34.1 ± 3.2^c^
Total LCPUFAs	21.3 ± 1.8	21.0 ± 1.6	14.8 ± 0.5	19.9 ± 1.7
Total n-6 LCPUFAs	12.4 ± 1.2	11.7 ± 1.1	11.4 ± 0.9	12.4 ± 1.3
Total n-3 LCPUFAs	8.90 ± 0.5^c,d^	9.30 ± 0.7^c,d^	3.40 ± 0.2^a,b,d^	7.50 ± 0.3^a,b,c^
n-6/n-3 LCPUFA ratio	1.39 ± 0.2^c^	1.26 ± 0.2^c^	3.35 ± 0.2^a,b,d^	1.65 ± 0.3^c^

Values are expressed as g fatty acid per 100 g FAME and represent the mean ± SEM for *n* = 12–14 mice per experimental group. Significant differences between the groups are indicated by the letter identifying each group (one-way ANOVA and Newman-Keuls´ post-test; *p* < 0.05). The identification of saturated and unsaturated fatty acids and their relationships are shown in Table 2

The FA composition of testicle phospholipids was modified by HFD and HT interventions (Table 6), as observed in the other studied tissues. HT supplementation had no effect in the control groups, but it restored the values of ALA, EPA, DPA (n-6), DPA (n-3), and DHA, which were significantly reduced by the HFD, to figures close to the control values. HFD increased total SFA and n-6/n-3 LCPUFA ratio and decreased total LCPUFAs and total n-3 LCPUFAs, values that were also restored to control levels by HT supplementation.

**Table 6:** Fatty acid composition of testicle phospholipids from control mice and high-fat diet (HFD) fed mice receiving hydroxytyrosol (HT) supplementation

	Fatty acid composition (g/100 g FAME)			
	Groups			
	Control diet (CD)		High fat diet (HFD)	
	Saline	HT	Saline	HT
Fatty acid	(a)	(b)	(c)	(d)
C16:0	30.6 ± 3.0^c^	29.2 ± 2.7^c^	41.6 ± 3.9^a,b,d^	29.5 ± 2.9^c^
C18:0	7.51 ± 1.2	6.99 ± 0.8	6.21 ± 1.1	7.44 ± 0.9
C18:1n-9	22.6 ± 2.0	23.9 ± 2.4	21.8 ± 1.9	24.5 ± 2.6
C18:2n-6 (LA)	11.6 ± 0.6	12.8 ± 0.9	10.6 ± 0.6	11.2 ± 0.5
C18:3n-3 (ALA)	2.06 ± 0.2^c^	2.21 ± 0.2^c^	0.56 ± 0.05^a,b,d^	1.98 ± 0.1^c^
C20:4n-6 (AA)	8.54 ± 0.7	9.05 ± 0.9	8.40 ± 0.8	8.38 ± 0.7
C20:5n-3 (EPA)	1.19 ± 0.2^c^	1.36 ± 0.1^c^	0.46 ± 0.05^a,b,d^	1.25 ± 0.2^c^
C22:5n-6 (DPAn-6)	0.38 ± 0.06^c^	0.45 ± 0.07^c^	0.12 ± 0.03^a,b,d^	0.41 ± 0.06^c^
C22:5n-3 (DPAn-3)	0.44 ± 0.05^c^	0.49 ± 0.06^c^	0.15 ± 0.02^a,b,d^	0.43 ± 0.07^c^
C22:6n-3 (DHA)	10.2 ± 1.1^c^	10.6 ± 1.4^c^	4.58 ± 0.8^a,b,d^	9.36 ± 1.2^c^
Total SFAs	38.9 ± 3.3^c^	36.8 ± 3.1^c^	48.2 ± 4.1^a,b,d^	38.4 ± 3.4^c^
Total MUFAs	25.7 ± 2.5	26.6 ± 2.2	24.9 ± 2.1	27.6 ± 2.4
Total PUFAs	35.4 ± 3.1^c^	36.6 ± 3.4^c^	26.9 ± 2.2^a,b,d^	34.0 ± 3.0^c^
Total LCPUFAs	21.1 ± 1.9^c^	22.5 ± 2.2^c^	13.9 ± 1.0^a,b,d^	20.5 ± 2.0^c^
Total n-6 LCPUFAs	9.12 ± 0.8	9.70 ± 0.9	8.70 ± 0.7	9.00 ± 0.8
Total n-3 LCPUFAs	11.9 ± 1.1^c^	12.8 ± 1.4^c^	5.20 ± 0.5^a,b,d^	11.5 ± 0.9^c^
n-6/n-3 LCPUFA ratio	0.77 ± 0.05^c^	0.76 ± 0.04^c^	1.67 ± 0.3^a,b,d^	0.78 ± 0.04^c^

Values are expressed as g fatty acid per 100 g FAME and represent the mean ± SEM for *n* = 12–14 mice per experimental group. Significant differences between the groups are indicated by the letter identifying each group (one-way ANOVA and Newman-Keuls´ post-test; *p* < 0.05). The identification of saturated and unsaturated fatty acids and their relationships are shown in Table 2

## Discussion

The liver has a central role in the synthesis of n-6 and n-3 LCPUFAs, this tissue being responsible for the majority of this conversion through the processes of desaturation and elongation of AL and ALA to the respective metabolic products [34]. The role of Δ-5 and Δ-6 desaturases in this process is highly regulated by hormones, particularly by insulin-mediated upregulation of the gene expression of the enzymes [1, 10, 15]. However, several conditions can significantly diminish the capacity of the liver for LCPUFA synthesis from their precursors thus affecting the hepatic levels of n-6 n-3 LCPUFAs, including (i) the lack of nutrients such as zinc [35]; (ii) the excessive ethanol consumption [36]; and (iii) the existence of certain polymorphisms in the gene sequences of Δ-5 and Δ-6 desaturases that reduces the activity of these enzymes [37]. An interesting situation related to the activity of Δ-5 and Δ-6 desaturases is liver steatosis, a pathological condition in which the oxidative stress of nutritional origin generated by fat overload (lipotoxicity) also results in a reduction in the desaturation capacity of the tissue with low levels of n-6 and n-3 LCPUFAs [14, 38, 39], effects that are also observed after a HFD [15, 40]. Also, in overfeeding conditions the increment in gene expression of Δ-5 and Δ-6 desaturase enzymes is not sufficient to compensate the reduction of n-6 and n-3 LCPUFAs [39].

Data presented show that HFD induced significant metabolic changes in mice including (i) insulin resistance, evidenced by the enhancement in the HOMA index, concomitantly with increases in serum triacylglycerol’s and cholesterol levels; (ii) a rise in plasma and liver oxidative stress status involving a diminution in the activity of antioxidant enzymes and derangement in the GSH system; and (iii) a significant reduction in the synthesis and storage of n-6 and n-3 LCPUFAs in the studied tissues. The substantial diminution in the hepatic levels of LCPUFAs by HFD, particularly those from the n-3 series (EPA, DHA and DPA), may be ascribed to the drastic reduction of the liver activity of desaturase enzymes achieved, considering their relevance in LCPUFA metabolism [15, 40]. This effect can be contributed by the diminution in the hepatic levels of the n-3 LCPUFA precursor ALA observed (Table 2) and by the enhancement in the oxidative stress status of the liver (Fig. 3) that can elicit desaturase inactivation. The latter suggestion is based on the finding that oxidative stress triggers the carbonylation and misfolding of proteins, thus inducing endoplasmic reticulum (ER) stress [41, 42]. ER stress is a response that can be caused by the excess of palmitic acid (C16:0) induced by the HFD (Table 2), a major metabolite responsible for generating pro-oxidant status and lipotoxicity in the liver directly promoting protein folding [43]. The association of liver desaturation capacity and oxidative stress is supported by the significant direct correlation found between desaturase activity and the glutathione status and the inverse relationship concerning desaturase activity and the lipid peroxidation potential (Fig. 4). Despite the HFD-induced downregulation of Δ-5 and Δ-6 desaturase activity, the mRNA expression of both desaturases was upregulated, a finding that agrees with the enhancement in the mRNA expression of SREBP-1c and its DNA binding capacity, a lipogenic transcription factor mediating the transcriptional activation of desaturase genes by insulin and inhibition by LCPUFAs [20, 44]. Although upregulation of SREBP-1c by insulin is unlikely to occur under insulin resistance conditions, SREBP-1c induction by HFD could be accomplished by ER-stress, which, in addition, upregulates the expression of other lipogenic factors including C/EBP and peroxisome proliferator-activated receptor-γ (PPAR-γ) [45]. Furthermore, genetic ablation of solute carrier family 7a3a mediating arginine transport for nitric oxide (NO) biosynthesis also leads to hepatic steatosis in liver cells from zebrafish, mice, and humans, through downregulation of NO-dependent AMPK-PPAR-α signaling [46]. However, additional studies are required to assess the involvement of Slc7a3a in HFD-induced liver steatosis.

A direct consequence of HFD-induced decrease in the desaturase activity of the liver, with reduction in the synthesis of LCPUFAs, is the diminution of the n-6 and n-3 LCPUFA levels in red blood cells, brain, heart, and testicle, considering that the liver is the main organ for LCPUFA biosynthesis in mammals [9, 10]. In this context, it is important to note that the LCPUFA content in erythrocytes is considered a metabolic marker of the liver synthesis of n-6 and n-3 LCPUFAs from their respective precursors [10, 14, 15, 38, 47]. Moreover, reduction in the hepatic synthesis and transport of LCPUFAs to extrahepatic tissues may have a direct impact upon brain structure and function [48], in the appropriate functioning of heart cell membranes, representing a main mechanism supporting the cardio-protective properties of n-3 LCPUFA [49], and in sperm viability, considering that DHA is mainly accumulated into sperms having a relevant role in fertilization [50–52]. For example when the contribution of dietary LCPUFA is low, the synthesis of these fatty acids starting from their 18 carbon atoms precursors is increased in a significant form [53]. In this regard, when the diet not supply DHA, the synthesis of this fatty acid in the brain starting from its precursor increases up to 100 times [54].

Dietary supplementation with HT in animals fed a HFD achieved a significant reduction of metabolic disorders caused by the HFD, including the recovery of the desaturase activity of the liver and the LCPUFAs profile of liver and extrahepatic tissues, the prevention of the damage induced by oxidative stress being one of the most important benefits that can be outlined (Fig. 3). In this regard, the antioxidant properties derived from the HT has proven to exert effective hepatoprotection, either against lipid peroxidation, liver steatosis, ischemia/reperfusion injury, and inflammation [19, 55, 56]. Moreover, HT restored the intestinal barrier integrity and functions in mice fed high fat diet [55]. It is also possible that HT can modulate the absorption of lipids in the intestine, especially cholesterol [57], but this effect still requires further investigation. HT-induced enhancement in the antioxidative potential of the liver seems to be associated with activation of nuclear factor-erythroid related factor 2 (Nrf2), a transcription factor controlling the expression of SOD, CAT, GR, and GPX (Fig. 3), besides that of glutathione-S-transferase and detoxicant enzymes [58–61], with recovery of the glutathione status presumably being related to upregulation of the biosynthetic enzymes of the tripeptide. These redox-mediated protective effects of HT are complemented by reductions in SREBP-1c expression, mitochondrial abnormalities, and apoptosis [19], besides diminution of ER stress induced by tunicamycin in human liver cells [62], with suppression of cell growth in human hepatocellular carcinoma cells via inactivation of AKT and nuclear factor-κB (NF-κB) pathways [63]. The protective effects of HT are not restricted to the liver as this polyphenol relieves brain damage produced by subarachnoid hemorrhage in rats by preventing oxidative stress and reducing the activity of NF-κB [64], effects that are also evident in the prevention of neuronal damage induced by dopamine and 6-hydroxydopamine through an enhanced expression of phase II-detoxification enzymes such as NADPH quinone oxidoreductase 1 [65].

## Conclusions

Dietary supplementation with HT mitigates the deleterious metabolic effects produced by HFD in mice. Protective effects of HT in the liver are associated to (i) the recovery of the activity of Δ-5 and Δ-6 desaturase enzymes, with prevention of n-3 LCPUFAs depletion, (ii) a reduction in the oxidative stress status; (iii) downregulation of the lipogenic factor SREBP-1c; and (iv) the maintenance of the levels of n-3 LCPUFAs in extrahepatic tissues. In this respect, it was hypothesized that n-3 LCPUFA-aspirin combined protocols may improve the management of sepsis and acute respiratory distress syndrome, due to the n-3 LCPUFA-derived resolvins generation that are potent anti-inflammatory mediators [66]. In regard to the dose of HT used in our study (5 mg/kg/day) and the protective effects generated in mice fed with HFD, other researchers have used higher levels of HT (10 mg/kg/day) [19, 56]. In the future, these aspects need more investigations. Data presented also demonstrate the importance of dietary interventions that consider supplementation with HT, particularly due to its antioxidant potential, observation that reinforces the importance of dietary interventions addressing oxidative stress prevention and n-3 LCPUFA tissue level preservation, thus strengthening the contention that many of the benefits associated to the consumption of EVOO may be associated to its content of HT [39].

## Additional file

Additional file 1: Table S1. Composition of the experimental diets. **Table S2.** Gene specific primer sequences used in the study. (DOC 52 kb)

## Abbreviations

AA: Arachidonic acid
ALA: Alpha-linolenic acid
ALT: Alanine transaminase
AST: Aspartate transaminase
BHT: Butylated hydroxytoluene
CAT: Catalase
CD: Control diet
COX1: Cyclooxygenase 1
DHA: Docosahexaenoic acid
DHGLA: Dihomo-gamma-linolenic acid
EPA: Eicosapentaenoic acid
EVOO: Extra virgin olive oil
FAME: FA methyl esters
FAs: Fatty acids
FID: Flame ionization detector
GLA: Gamma-linolenic acid
GPX: Glutathione peroxidase
GR: Glutathione reductase
GSGG: Glutathione disulfide
GSH: Reduced glutathione
HFD: High-fat diet
HOMA: Homeostasis model assessment method
HT: Hydroxytyrosol
LA: Linoleic acid
LCPUFAs: Long-chain polyunsaturated fatty acids
NF-κB: Nuclear factor-κB
NO: Nitric oxide
Nrf2: Nuclear factor-erythroid related factor 2
PPAR-γ: Peroxisome proliferator-activated receptor-γ
PUFA: Polyunsaturated fatty acids
ROS: Reactive oxygen species
SOD: Superoxide dismutase
SREBP1c: Sterol regulatory element-binding protein 1c
TBARs: Thiobarbituric acid reactants
TLC: Thin layer chromatography
