# Supplementary material for: Hydroxytyrosol prevents reduction in liver activity of Δ-5 and Δ-6 desaturases, oxidative stress, and depletion in long chain polyunsaturated fatty acid content in different tissues of high-fat diet fed mice
Source: Lipids Health Dis. 2017 Apr 11;16:64. doi: 10.1186/s12944-017-0450-5 (PMC5387240; doi:10.1186/s12944-017-0450-5)
Supplement: Additional file 1: Table S1. — Composition of the experimental diets. Table S2. Gene specific primer sequences used in the study. (DOC 52 kb) [file 12944_2017_450_MOESM1_ESM.doc]

Additional file 1

Table S1. Composition of the experimental diets

| Product | CD (#D12450K) | | HFD (#D12492) | |
| --- | --- | --- | --- | --- |
|  | gm% | kcal% | gm% | kcal% |
| Protein | 19.2 | 20 | 26.2 | 20 |
| Carbohydrate | 67.3 | 70 | 26.3 | 20 |
| Fat | 4.3 | 10 | 34.9 | 60 |
| Total | - | 100 | - | 100 |
| kcal/gm | 3.85 | - | 5.24 | - |
| Ingredient | gm | kcal | gm | kcal |
| Casein, 30 Mesh | 200 | 800 | 200 | 800 |
| L-Cystine | 3 | 12 | 3 | 12 |
| Corn Starch | 550 | 2200 | 0 | 0 |
| Maltodextrin 10 | 150 | 600 | 125 | 500 |
| Sucrose | 0 | 0 | 68.8 | 275.2 |
| Cellulose, BW200 | 50 | 0 | 50 | 0 |
| Soybean Oil | 25 | 225 | 25 | 225 |
| Lard* | 20 | 180 | 245 | 2205 |
| Mineral Mix S10026 | 10 | 0 | 10 | 0 |
| DiCalcium Phosphate | 13 | 0 | 13 | 0 |
| Calcium Carbonate | 5.5 | 0 | 5.5 | 0 |
| Potassium Citrate, 1 H_2_O | 16.5 | 0 | 16.5 | 0 |
| Vitamin Mix V10001 | 10 | 40 | 10 | 40 |
| Choline Bitartrate | 2 | 0 | 2 | 0 |
| FD&C Red Dye #40 | 0.025 | 0 | - | - |
| FD&C Blue Dye #1 | 0.025 | 0 | 0.05 | 0 |
| Total | 1055.05 | 4057 | 773.85 | 4057 |

* CD#D12450K

Typical analysis of cholesterol in lard = 0.72 mg/gram.

Cholesterol (mg)/4057 kcal = 14. 4 Cholesterol (mg)/kg 13.6

* HFD#D12492:

Typical analysis of cholesterol in lard = 0.72 mg/gram.

Cholesterol (mg)/4057 kcal = 216. 4 Cholesterol (mg)/kg 279.6

Table S2. Gene specific primer sequences used in the study.

| mRNA | Forward primer | Reverse primer |
| --- | --- | --- |
| Δ-5 desaturase | GATGAACCATATCCCCATGC | TTGGCGCACAGGCATTG |
| Δ-6 desaturase | GGAACCATCGACATTTCCAG | TCTTTATGTCGGGGTCCTTG |
| SREBP-1c | CTGGAGACATCGCAAACAAGC | ATGGTAGACAACAGCCGCATC |
| β-actin | ACTGCCGCATCCTCTTCCTC | CTCCTGCTTGCTGATCCACATC |

Sequences are listed in the 5` → 3` direction.
